# Development and usefulness of an immunochromatographic device to detect antibodies for rapid diagnosis of human gnathostomiasis

**DOI:** 10.1186/s13071-016-1294-y

**Published:** 2016-01-12

**Authors:** Penchom Janwan, Pewpan M. Intapan, Hiroshi Yamasaki, Rutchanee Rodpai, Porntip Laummaunwai, Tongjit Thanchomnang, Oranuch Sanpool, Kaoru Kobayashi, Katsuyoshi Takayama, Yukuharu Kobayashi, Wanchai Maleewong

**Affiliations:** Research and Diagnostic Center for Emerging Infectious Diseases, Faculty of Medicine, Khon Kaen University, Khon Kaen, 40002 Thailand; Department of Medical Technology, School of Allied Health Sciences and Public Health, Walailak University, Nakhon Si Thammarat, 80161 Thailand; Department of Parasitology, Faculty of Medicine, Khon Kaen University, Khon Kaen, 40002 Thailand; Department of Parasitology, National Institute of Infectious Diseases, Tokyo, 162-8640 Japan; Faculty of Medicine, Mahasarakham University, Mahasarakham, 44000 Thailand; Division of Research and Development, Adtec Inc., Oita, 879-0471 Japan

**Keywords:** *Gnathostoma spinigerum*, Human gnathostomiasis, Immunochromatographic test kit, Serodiagnosis, Recombinant protein

## Abstract

**Background:**

Human gnathostomiasis is a serious tropical disease, which is often overlooked. There is an urgent need to improve tools to aid the potential diagnosis of the disease in endemic regions. To overcome this, we produced the immunochromatographic test (ICT) kit for a rapid and simple diagnosis of human gnathostomiasis.

**Findings:**

The recombinant protein (named rGslic18) was applied to ICT kit as the antigen. The diagnostic value of ICT kit was evaluated using serum samples from parasitologically proven and clinically suspected gnathostomiasis patients, healthy volunteers and patients with other parasitic diseases. The ICT kit exhibited quite high sensitivity (93.75 %) and specificity (97.01 %).

**Conclusions:**

The ICT kit is simple, convenient and easy to implement and expected to provide reliable diagnostic results for human gnathostomiasis. It also will be a promising diagnostic tool not only for large-scale epidemiological surveys in endemic or remote areas where diagnostic facilities are poor but also for a rapid clinical diagnosis in the bedside laboratory.

## Background

Human gnathostomiasis is a serious food-borne parasitic zoonosis caused by infections with larval spirurid nematodes *Gnathostoma* spp. and the disease is regularly found in Asia and the Americas [1]. It is also common in travelers returning from visits to areas endemic to this harmful parasite [1]. Definitive diagnosis for human gnathostomiasis can be made by detecting the larvae migrating out from the human body. However, since direct detection of the parasite is difficult and often unsuccessful, diagnosis of human gnathostomiasis is primarily made by relying upon clinical features, history of eating parasite-contaminated food, elevated blood eosinophilia, and serological outcomes [1]. Here, a recombinant protein produced from a *Gnathostoma spinigerum* clone (named Gslic18) isolated from *G. spinigerum* cDNA library, was used as an alternative antigen for the immunochromatographic test (ICT) kit development. The result was analysed and compared with specific IgG antibody detection against the native 24/21 kDa *G. spinigerum* larval antigen using immunoblotting [2]. This study demonstrated the effectiveness of the ICT kit as a convenient and rapid platform in the diagnosis of human gnathostomiasis that had not yet been reported elsewhere.

## Methods

*Gnathostoma spinigerum* advanced third-stage larvae RNA (920 ng) was used for cDNA synthesis. The cDNA library was constructed and colonies showing positive immunoscreening with serum samples from gnathostomiasis patients were selected for cloning procedure. A clone (named Gslic18) with a strong positive reaction to IgG antibodies in serum samples from gnathostomiasis patients, but not reactive to serum samples from patients with other parasitic infections or those in the negative control group, was selected through immunoscreening. The cDNA insert of Gslic18 was subcloned into a pQE-31 expression vector and transformed into *Escherichia coli* XL-1 Blue (Qiagen, Hilden, Germany) as the expression system. The recombinant Gslic18 (rGslic18) protein was expressed as insoluble protein and solubilised using urea solution (8 M urea, 0.1 M NaH_2_PO_4_, 0.01 M Tris-HCl, pH 8.0). Then, the rGslic18 carrying 6-Histidine (6-His)-tagged residues was purified using Ni-NTA His Bind Resin (Novagen, Darmstadt, Germany) according to the manufacturer’s protocol. The purified protein concentration was determined [3]. All human serum samples used for diagnostic values were supplied by the frozen bank (-70 °C). The samples were divided into three groups: (i) the negative control group (*n* = 20) comprised samples from healthy adult volunteers who underwent stool examination [4] and were found to be free from any intestinal parasitic infection at the time of blood collection (a pooled serum consisting of samples from the healthy individuals was also used as a negative control for each assay); (ii) the gnathostomiasis group (*n* = 32), which included samples from parasitologically confirmed gnathostomiasis patients (*n* = 9) and from patients showing clinical symptoms of suspected cutaneous and visceral gnathostomiasis, and neurognathostomiasis (*n* = 23) [1] with a history of eating food possibly contaminated with *Gnathostoma* larvae and were positive 24/21 kDa *G. spinigerum* antigen by immunoblotting [2]; and (iii) the third group (*n* = 114), which consisted of serum samples from patients with parasitic infections other than gnathostomiasis. Their infections were confirmed by parasitological methods except in cases of cysticercosis which were diagnosed by a computerised tomography scan and found positive by the immunological method [5] (Table 1).

**Table 1:** Types of human sera examined and diagnostic results of the ICT kit and the immunoblotting using the 24/21 kDa *G. spinigerum* antigen

Type of serum samples	Number of positive/Total number	
	ICT kit	Immunoblotting
Healthy control	0/20	0/20
Confirmed gnathostomiasis	9/9	9/9
Suspected gnathostomiasis	21/23	23/23
Cysticercosis	1/4	0/4
Taeniasis	0/10	0/10
Opisthorchiasis viverrini	0/15	0/15
Fascioliasis	1/5	0/5
Paragonimiasis	1/10	0/10
Angiostrongyliasis	1/10	0/10
Strongyloidiasis	0/10	0/10
Hookworm infection	0/10	0/10
Capillariasis	0/10	0/10
Ascariasis	0/10	0/10
Trichinellosis	0/3	0/3
Sparganosis	0/2	0/2
Malaria	0/10	0/10
Filariasis	0/5	0/5

**Fig. 1:**
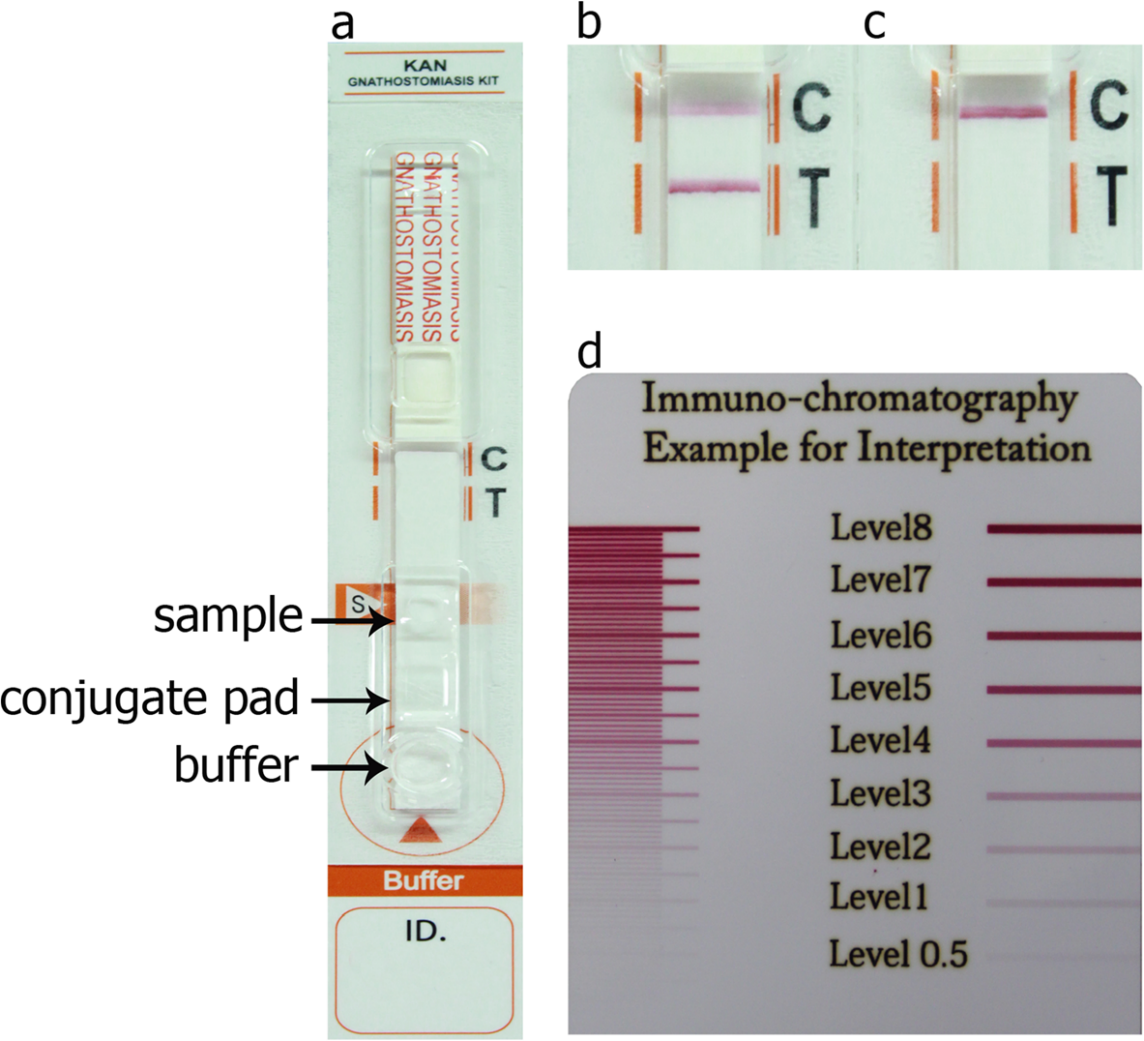
The KAN gnathostomiasis kit (**a**) with immunochromatographic assay for the diagnosis of human gnathostomiasis. Representative images of ICT strips on which positive (**b**) and negative (**c**) results are shown. Each diluted serum sample was dropped onto the inscription “sample”, and a buffer was applied onto inscription “buffer”. A criterion of the diagnostic result is whether a red band appears at the test (T) line within 15 minutes or not. In the positive serum sample, the test (T) line and control (C) line turned red (**b**) but in the negative serum sample, only the control (C) line turned red (**c**). The intensity of the bands was estimated visually (unaided) according to the reference board (**d** considering level 2 as the cutoff level)

The rapid ICT kit (named the “KAN gnathostomiasis kit, K, Khon Kaen University; A, Adtec Inc. Oita; N, National Institute of Infectious Diseases, Tokyo”) using the rGslic18 antigen was optimised based on the ELISA result using the same antigen at Adtec Inc., Oita, Japan. One mg/ml of anti-human IgG (H + L) (Medical and Biological Laboratories Co., Ltd., Nagoya, Japan) was absorbed at control line (C), 0.5 mg/ml of the rGslic18 antigen was absorbed at test line (T), and OD 1.0 of colloidal gold conjugated with anti-human IgG (Adtec Inc.) was sprayed on a piece of glass fiber (conjugate pad) (Fig. 1). The kit consists of an immunochromatographic device, sample buffer for diluting serum sample and buffer for chromatography. The diagnostic procedure is as follows: dilute serum samples with sample buffer in 1:50 and spot the aliquot (5 μl) onto inscription “sample”, and apply buffer (60 μl) onto inscription “buffer” (Fig. 1a). A criterion of the diagnostic result is whether a red band appears at the test line within 15 min or not. The intensity of the bands was estimated visually (unaided) according to the reference board (Fig. 1d, with level 2 as the cutoff level). Positive results were judged by the appearance of red band at the test (T) line and the control (C) line (Fig. 1b). Negative results were judged by the disappearance of a red band at test (T) line and appearance at control (C) line (Fig. 1c). The diagnostic parameters of sensitivity and specificity were computed as previously [6]. The diagnostic values of the ICT kit were evaluated with those of immunoblotting [2] and percentages of total concordance were computed (percent concordance (%) = (the number of concordances between ICT and the immunoblotting reference × 100)/the number of tested samples). Informed consent was obtained from all human adult participants and from parents or legal guardians of minors. The study protocol was accepted by the Khon Kaen University Ethics Committee for Human Research (HE571438).

## Results and discussion

**Fig. 2:**
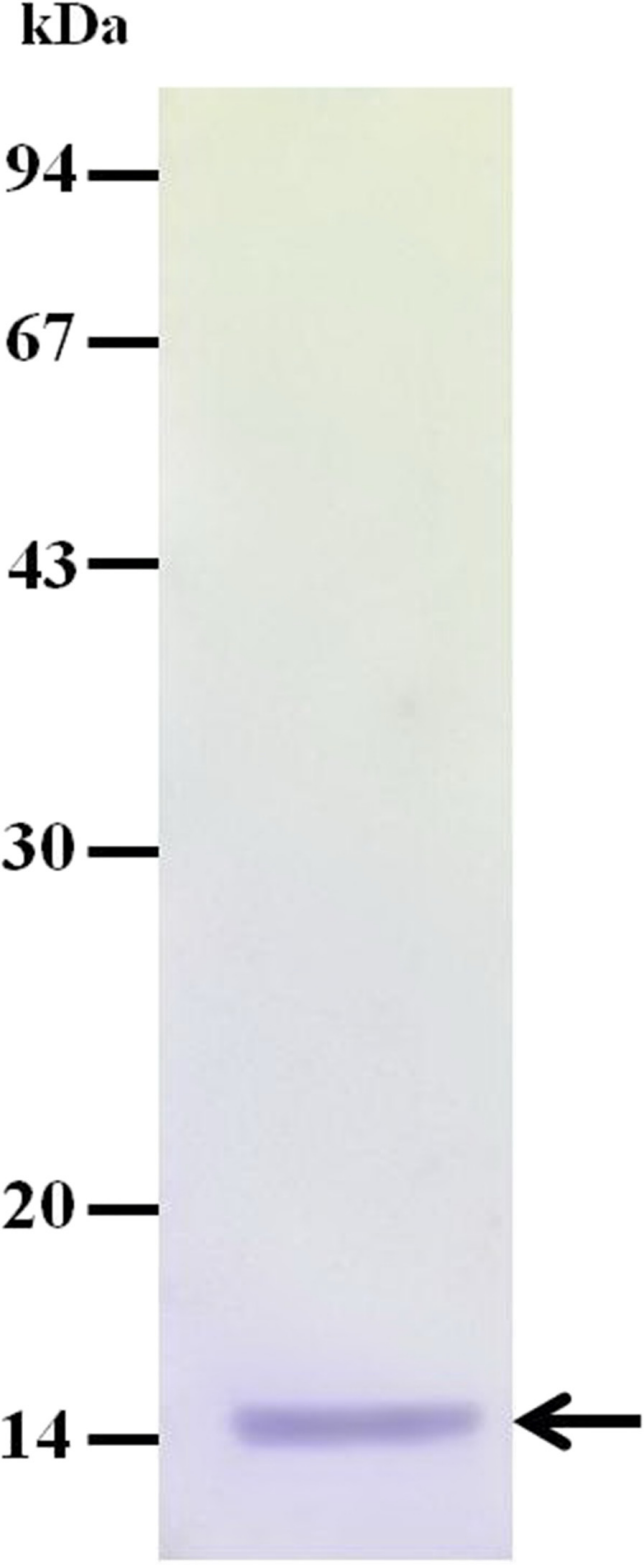
The purified rGslic18 protein fused with His-tagged residues on 12 % sodium dodecyl sulfate polyacrylamide gel electrophoresis analysis. The protein was stained with Coomasie Brilliant Blue. An arrow indicates band of the rGslic18 protein (14 kDa)

The purified rGslic18 protein used as an antigen shown in Fig. 2 was used for preparation of immunochromatographic strips. The KAN gnathostomiasis kit, which employed the diagnostic values of the purified rGslic18 protein for human gnathostomiasis, was evaluated using serum from individuals among the gnathostomiasis patients, the healthy control, and the patients with other parasitic diseases (Table 1). All serum samples from the confirmed and suspected (except 2 cases) gnathostomiasis patients yielded positive results. In contrast, none of the 20 healthy control sera showed positive results. Some cross-reactivity was observed in serum samples of cysticercosis (1 of 4), fascioliasis (1 of 5), paragonimiasis (1 of 10), and angiostrongyliasis (1 of 10). The ICT kit exhibited high sensitivity (93.75 %) and specificity (97.01 %). Table 2 summarises the comparison of immunoblotting using immunodiagnostic 24/21 kDa *G. spinigerum* antigen and the ICT kit using the sera from the 166 subjects. Using the 24/21 kDa *G. spinigerum* antigen with immunoblotting as a standard test, both tests detected 30 positive cases and 130 negative cases. There were four false positive tests and two false negative tests detected by the KAN gnathostomiasis kit. The concordance of the ICT kit was determined to be 96.39 % (160/166 sera).

**Table 2:** Comparison among ICT kit using rGslic18 and immunoblotting using 24/21 kDa *G. spinigerum* antigen

Test type and results	Immunoblotting		
ICT kit^a^	No. positive	No. negative	Total
No. positive	30	4	34
No. negative	2	130	132
Total	32	134	166

^a^ There was no significant difference with immunoblotting (*p* > 0.05; Exact McNemar's test)

## Conclusions

This is the first time that the ICT kit with rGslic18 as an antigen was applied for the detection of anti-*Gnathostoma* antibodies in human sera and the results revealed high sensitivity and specificity. The ICT kit is a fast, simple, easy to implement and reliable as previous reports [7–9]. It has the potential to aid the development of a promising serodiagnostic kit for human gnathostomiasis as a stable mass production system, particularly for screening patients on a massive-scale in endemic areas or clinical diagnosis in the laboratory.
